# Correlation Between Components of Malnutrition Diagnosed by Global Leadership Initiative on Malnutrition Criteria and the Clinical Outcomes in Gastric Cancer Patients: A Propensity Score Matching Analysis

**DOI:** 10.3389/fonc.2022.851091

**Published:** 2022-03-03

**Authors:** Li-Bin Xu, Ting-Ting Mei, Yi-Qi Cai, Wen-Jing Chen, Si-Xin Zheng, Liang Wang, Xiao-Dong Chen, Yun-Shi Huang

**Affiliations:** ^1^ Department of Gastrointestinal Surgery, The First Affiliated Hospital, Wenzhou Medical University, Wenzhou, China; ^2^ Department of Gastrointestinal Surgery, Affiliated Hangzhou First People’s Hospital, Zhejiang University School of Medicine, Hangzhou, China; ^3^ Department of Trauma & Emergency Surgery, The First Affiliated Hospital, Wenzhou Medical University, Wenzhou, China

**Keywords:** malnutrition, gastric cancer, clinical outcomes, propensity score matching, global leadership initiative on malnutrition

## Abstract

**Objective:**

Malnutrition is recognized as a risk factor for poor outcome in patients with gastric cancer (GC). In 2018, the Global Leadership Initiative on Malnutrition (GLIM) published standardized criteria for the diagnosis of malnutrition. Our aim was to investigate whether any of the components of the GLIM diagnostic criteria were related to worse clinical outcomes in patients with GC.

**Methods:**

This study analyzed patients with GC who underwent radical gastrectomy in our hospital between 2014 and 2019. A preoperative nutritional assessment was performed for each patient. Matching was based on the presence of three GLIM components: high weight loss (WL), low body mass index (BMI), and low skeletal muscle index (SMI).

**Results:**

The analysis included 1,188 patients, including 241 (20.3%) with high WL, 156 (13.1%) with low BMI, and 355 (29.9%) with low SMI. Before matching, patients who met the GLIM component criteria were mostly associated with older age, low nutritional reserves, and late tumor progression. After matching, the clinical characteristics of the three cohorts were balanced. In the matched queue, the survival prognosis of the high WL group was worse than that of the non-WL group, and the postoperative complication rate was higher in the low SMI group than in the normal SMI group (P <0.05). In addition, the clinical outcomes in the low and normal BMI groups were similar (P >0.05).

**Conclusion:**

Of the GLIM criteria, high WL and low SMI may be associated with poor clinical outcomes in patients with GC, while a low BMI may not be associated with outcome.

## Introduction

In 2020, the estimated number of new global cases of gastric cancer (GC) was more than one million, ranking fifth of all newly diagnosed cancers (1). As a malignant tumor of the upper digestive tract, GC often aggressively impacts the nutritional status of the patient. According to the previous different definitions of malnutrition, the prevalence in GC patients ranges from 20.9% to 80.4% (2–4). GC patients often experience tumor-related symptoms, such as cancer pain, anorexia, and malabsorption, and additional malnutrition may be induced by efforts to treat the patient, e.g., the gastrectomy itself and the side effects of adjuvant chemotherapy. Approximately 20% of cancer patients die from cachexia rather than from the malignant tumor itself (5). Timely detection of malnutrition and effective nutritional interventions have been proven to have considerable clinical and financial benefits (6, 7).

Many methods for nutritional screening and evaluation have been used to determine the nutritional status of patients, but there is no current consensus on the diagnostic criteria for malnutrition. The Global Leadership Initiative on Malnutrition (GLIM) has published a new definition of adult malnutrition to establish a global consensus concerning the diagnostic criteria of malnutrition in clinical diagnosis and treatment (8). After ranking the GLIM criteria, five core diagnostic criteria were screened, including three phenotypic criteria and two etiological criteria. To date, some studies have demonstrated the effectiveness of the GLIM criteria in nutritional assessment and prognosis prediction in adult cancer patients (9–11). However, to the best of our knowledge, only a few studies have explored the correlation between the different components of GLIM and the clinical outcomes of patients in medical institutions (12, 13). Studies have shown that malnutrition defined by GLIM criteria is associated with poor prognosis in GC patients (14, 15), whereas evidence for the correlation of GLIM components with GC prognosis is still insufficient. Therefore, this study aimed to explore the correlation between any GLIM phenotypic criteria, survival time, and postoperative complications in patients with GC.

## Materials and Methods

### Participants

**Figure 1:**
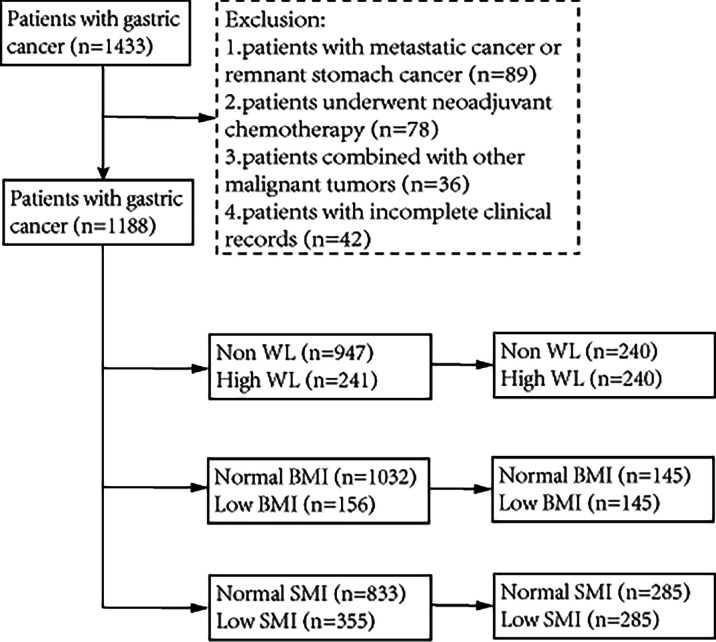
The flowchart of patient inclusion.

A total of 1,433 patients with GC who underwent radical gastrectomy between July 2014 and February 2019 at the First Affiliated Hospital of Wenzhou Medical University were recruited for this retrospective cohort study. The inclusion criteria were as follows: (a) pathologically diagnosed primary gastric adenocarcinoma before surgery; and (b) planned radical gastrectomy. The exclusion criteria were as follows: (a) patients with metastatic cancer or remnant stomach cancer; (b) patients who underwent neoadjuvant chemotherapy; (c) patients with other malignant tumors; and (d) patients with incomplete clinical records. Ultimately, 1,188 patients were included in the analysis. The management of GC was mainly based on the Japanese Gastric Cancer Treatment Guidelines 2010 (16). Relevant departments were invited to develop individualized treatment plans for patients when necessary. In addition, malnutrition was not an index of avoiding surgical treatment for this study’s cohort. The detailed elimination process and group analysis are shown in **Figure 1**. All research data was obtained from the database collected by the institution prospectively and the inpatient electronic medical record system. This study was approved by the ethics committee of our hospital and complied with the Declaration of Helsinki. Considering the nature of retrospective research, the requirement for informed consent form was waived.

### Data Collection

The following data were collected and analyzed for all patients: (1) preoperative basic information, including sex, age, height, weight, hemoglobin concentration, albumin concentration, and comorbidities; (2) nutritional data, including weight loss (WL), body mass index (BMI), and skeletal muscle index (SMI); (3) postoperative pathology information, including tumor size, pathologic tumor–node–metastasis (pTNM) stage, and tumor differentiation degree; and (4) prognostic information, including postoperative complications (PC), postoperative length of stay (LOS), readmission rate within 1 month, and long-term survival information. Complications were classified in accordance with the Clavien–Dindo classification (17). Major PC was defined as a complication of grade II or above, while severe PC was defined as a complication of grade III or above.

Each patient underwent an outpatient follow-up examination in the first month after surgery. Afterwards, additional follow-ups were performed by phone or outpatient examination every 3–6 months. Each follow-up visit included a physical examination, laboratory examination, and necessary imaging examinations, such as computed tomography (CT), ultrasound, and endoscopy. Overall survival (OS) time was defined as the time from the date of surgery to death from any cause or the last follow-up visit. Disease-free survival (DFS) time was defined as the date of surgery to the date of tumor recurrence or death from any cause. The last follow-up was conducted in July 2021.

### Components of GLIM Criteria

The GLIM criteria have five components, including three phenotypic criteria (involuntary WL, low BMI, and low muscle mass) and two etiological criteria (reduced food intake or absorption, and disease burden or inflammation) (8). As a tumor of the digestive tract, GC is considered a chronic disease, and studies have shown that GC is closely associated with inflammation (18, 19). Therefore, all patients with GC in this study are considered to meet the etiological criteria of burden of disease/inflammation. Thus, this study aimed to explore the correlation between the three phenotypic criteria and the prognosis of patients with GC. Involuntary WL was calculated as the weight change in GC patients before surgery. According to the GLIM criteria (8), high WL was defined as a WL >5% within 6 months or >10% over 6 months. According to the GLIM recommendations for Asians (8), low BMI was defined as a BMI <18.5, when patients were <70 years old or <20 when patients were ≥70 years old.


$$SMI=\frac{{\text{total muscle area of L}3[\text{cm}}^{2}]}{{\text{height [m]}}^{2}}$$

Low SMI was defined as ≤40.8 cm^2^/m^2^ for males and ≤34.9 cm^2^/m^2^ for females according to the prospective study (21, 22).

### Statistical Analysis

The Kolmogorov–Smirnov test was used to determine the continuous data distribution state. Continuous data were expressed as mean ± standard deviation (SD) or median (interquartile range, IQR) according to the data distribution, and the differences between groups were compared using Student’s t-test or Mann-Whitney U test. Categorical data were expressed as counts and percentages and were compared using the chi-square test.

To reduce the influence of selection bias and potential confounding factors on the prognostic analysis, we performed propensity score matching (PSM) analysis to obtain matched data. Three different matches were used to explore the correlation between the three phenotypic criteria and prognoses of patients with GC. Propensity scores were calculated based on the following patient data: age, Charlson score, hemoglobin, albumin, tumor size, TNM stage, and two phenotypic indicators (BMI and SMI, WL and SMI, WL, and BMI). Three PSMs were conducted at a ratio of 1:1 using a caliper set to 0.20. Then, three sets of matched cohorts were obtained: the high and non-WL groups, the low and normal BMI groups, and the low and normal SMI groups. The Kaplan–Meier method was used to analyze the OS and DFS rates. The log-rank test was used to compare survival differences between the groups. Univariate and multivariate logistic and Cox regression analyses were used to analyze the risk factors of PCs and survival rates in the entire cohort. Factors with *P <*0.05 in the univariate analysis, were included in the multivariate analysis. Differences were considered statistically significant at *P <*0.05. All data were analyzed using SPSS Statistics (version 25.0; IBM Corp., Armonk, NY, USA) and R version 4.1.0 (http://www.r-project.org).

## Results

### Patient Characteristics

A total of 1,188 patients were included in the analysis, of which 867 (73.1%) were male. Baseline characteristics before matching are shown in **Table 1**. According to the GLIM criteria, 241 (20.3%) patients had high WL, 156 (13.1%) patients had low BMI, and 355 (29.9%) patients had low SMI. Before matching, patients who met the phenotypic criteria often had older age, anemia, hypoalbuminemia, and a more advanced tumor stage. After PSM analysis, there were 480 patients in the WL cohort (high WL group, n=240; non-WL group, n=240), 290 patients in the BMI cohort (low BMI group, n=145; normal BMI group, n=145), and 570 patients in the SMI cohort (low SMI group, n=285; normal SMI group, n=285). As shown in **Table 2**, the baseline characteristics of each group reached a balance after matching, except that female were more likely to have a low SMI.

**Table 1:** Patient baseline characteristics in the entire cohort.

Characteristic	All (n=1188)	GLIM-high WL			GLIM-low BMI			GLIM-low SMI		
		No (n=947)	Yes (n=241)	*P* value	No (n=1032)	Yes (n=156)	*P* value	No (n=833)	Yes (n=355)	*P* value
Sex, n (%)				0.236			0.546			0.002*
Female	319 (26.9)	247 (26.1)	72 (29.9)		274 (26.6)	45 (28.8)		202 (24.2)	117 (33.0)	
Male	869 (73.1)	700 (73.9)	169 (70.1)		758 (73.4)	111 (71.2)		631 (75.8)	238 (67.0)	
Age (years), median (IQR)	66 (58-73)	66 (58-73)	66 (59-73)	0.098	65 (58-72)	72 (63-78)	<0.001*	64 (57-71)	70 (62-76)	<0.001*
Weight loss (%), median (IQR)	0.0 (0.0-4.3)	0.0 (0.0-0.0)	9.0 (7.3-11.8)	<0.001*	0.0 (0.0-3.8)	0.0 (0.0-9.0)	<0.001*	0.0 (0.0-3.6)	0.0 (0.0-5.8)	0.001*
BMI (kg/m^2^), median (IQR)	22.5 (20.4-24.4)	22.8 (20.6-24.7)	21.5 (19.6-23.4)	<0.001*	23.0 (21.3-24.8)	18.2 (17.2-18.9)	<0.001*	23.4 (21.6-25.1)	20.3 (18.8-22.2)	<0.001*
SMI (cm^2^/m^2^), mean (SD)										
Female	37.2 (6.0)	37.3 (6.1)	36.7 (5.5)	0.440	37.8 (5.8)	33.3 (5.7)	<0.001*	40.6 (4.3)	31.2 (4.1)	<0.001*
Male	45.2 (7.6)	45.4 (7.6)	44.0 (7.5)	0.030*	46.3 (7.3)	37.8 (5.7)	<0.001*	48.6 (5.7)	36.1 (3.7)	<0.001*
Hemoglobin (g/L), median (IQR)	125 (105-138)	126 (109-139)	117 (96-134)	<0.001*	126 (108-139)	112 (93-127)	<0.001*	129 (112-140)	114 (94-128)	<0.001*
Albumin (g/L), median (IQR)	38.5 (35.1-41.3)	38.8 (35.3-41.6)	36.8 (34.0-40.0)	<0.001*	38.8 (35.6-41.5)	35.3 (32.6-39.2)	<0.001*	39.2 (36.0-41.7)	36.5 (33.4-39.7)	<0.001*
Charlson score, n (%)				0.441			0.032*			0.436
0	689 (58.0)	556 (58.7)	133 (55.2)		587 (56.9)	102 (65.4)		483 (58.0)	206 (58.0)	
1	293 (24.7)	226 (23.9)	67 (27.8)		255 (24.7)	38 (24.4)		212 (25.5)	81 (22.8)	
≥2	206 (17.3)	165 (17.4)	41 (17.0)		190 (18.4)	16 (10.3)		138 (16.6)	68 (19.2)	
Tumor size (cm), median (IQR)	3.5 (2.0-5.0)	3.0 (2.0-5.0)	4.5 (3.0-6.0)	<0.001*	3.4 (2.0-5.0)	3.5 (2.0-5.4)	0.433	3.0 (2.0-5.0)	4.0 (2.5-6.0)	<0.001*
pTNM stage, n (%)				<0.001*			0.037*			<0.001*
I	428 (36.0)	392 (41.4)	36 (14.9)		386 (37.4)	42 (26.9)		332 (39.9)	96 (27.0)	
II	249 (21.0)	192 (20.3)	57 (23.7)		210 (20.3)	39 (25.0)		165 (19.8)	84 (23.7)	
III	511 (43.0)	363 (38.3)	148 (61.4)		436 (42.2)	75 (48.1)		336 (40.3)	175 (49.3)	
Major PC, n (%)	297 (25.0)	227 (24.0)	70 (29.0)	0.104	241 (23.4)	56 (35.9)	0.001*	178 (21.4)	119 (33.5)	<0.001*
Severe PC, n (%)	115 (9.7)	82 (8.7)	33 (13.7)	0.018*	91 (8.8)	24 (15.4)	0.010*	65 (7.8)	50 (14.1)	0.001*
Postoperative LOS, median (IQR)	13 (11-18)	13 (11-17)	14 (11-18)	0.071	13 (11-17)	14 (11-20)	0.019*	13 (10-16)	14 (11-20)	<0.001*
Readmission, n (%)	71 (6.0)	54 (5.7)	17 (7.1)	0.429	62 (6.0)	9 (5.8)	0.907	49 (5.9)	22 (6.2)	0.834

GLIM, Global Leadership Initiative on Malnutrition; WL, weight loss; BMI, body mass index; SMI, skeletal muscle index; pTNM, pathologic tumor–node–metastasis; PC, postoperative complication; LOS, length of stay; IQR, interquartile range; SD, standard deviation.

^*^Statistically significant.

**Table 2:** Patient baseline characteristics in the propensity score-matched cohort.

Characteristic	GLIM-high WL			GLIM-low BMI			GLIM-low SMI		
	No (n=240)	Yes (n=240)	*P* value	No (n=145)	Yes (n=145)	*P* value	No (n=285)	Yes (n=285)	*P* value
Sex, n (%)			0.843			0.702			<0.001*
Female	74 (30.8)	72 (30.0)		46 (31.7)	43 (29.7)		59 (20.7)	101 (35.4)	
Male	166 (69.2)	168 (70.0)		99 (68.3)	102 (70.3)		226 (79.3)	184 (64.6)	
Age (years), median (IQR)	66 (60-74)	66 (59-73)	0.837	72 (62-76)	72 (61-78)	0.837	68 (61-74)	68 (61-75)	0.555
Weight loss (%), median (IQR)	0.0 (0.0-0.0)	9.0 (7.3-11.6)	<0.001*	0.0 (0.0-8.2)	0.0 (0.0-7.9)	0.715	0.0 (0.0-5.2)	0.0 (0.0-5.7)	0.262
BMI (kg/m^2^), median (IQR)	21.1 (19.5-23.4)	21.5 (19.6-23.4)	0.708	21.8 (20.4-24.8)	18.2 (17.2-18.9)	<0.001*	21.2 (19.9-22.5)	21.1 (19.4-22.8)	0.321
SMI (cm^2^/m^2^), mean (SD)									
Female	32.8 (4.9)	34.1 (5.5)	0.225	33.1 (4.6)	33.4 (5.7)	0.786	39.5 (4.2)	31.6 (2.8)	<0.001*
Male	43.6 (7.7)	44.1 (7.5)	0.561	38.2 (5.7)	38.3 (5.5)	0.960	46.4 (4.6)	36.5 (3.5)	<0.001*
Hemoglobin (g/L), median (IQR)	113 (95-130)	117 (96-134)	0.094	117 (94-130)	112 (93-127)	0.507	119 (94-134)	117 (99-130)	0.503
Albumin (g/L), median (IQR)	36.6 (33.4-39.7)	36.9 (34.0-40.0)	0.250	36.7 (33.6-40.0)	35.5 (32.8-39.4)	0.321	37.3 (34.0-40.3)	37.2 (34.2-42.9)	0.878
Charlson score, n (%)			0.273			0.572			0.467
0	148 (61.7)	132 (55.0)		98 (67.6)	94 (64.8)		170 (59.6)	168 (58.9)	
1	53 (22.1)	67 (27.9)		28 (19.3)	35 (24.1)		72 (25.3)	64 (22.5)	
≥2	39 (16.3)	41 (17.1)		19 (13.1)	16 (11.0)		43 (15.1)	53 (18.6)	
Tumor size (cm), median (IQR)	4.0 (3.0-6.0)	4.5 (3.0-6.0)	0.195	3.5 (2.3-5.0)	3.5 (2.0-5.0)	0.543	3.5 (2.0-5.5)	3.5 (2.0-6.0)	0.580
pTNM stage, n (%)			0.503			0.236			0.936
I	35 (14.6)	36 (15.0)		46 (31.7)	41 (28.3)		89 (31.2)	85 (29.8)	
II	47 (19.6)	57 (23.8)		26 (17.9)	38 (26.2)		62 (21.8)	63 (22.1)	
III	158 (65.8)	147 (61.3)		73 (50.3)	66 (45.5)		134 (47.0)	137 (48.1)	
Major PC, n (%)	69 (28.7)	69 (28.7)	1.000	39 (26.9)	50 (34.5)	0.161	68 (23.9)	89 (31.2)	0.049*
Severe PC, n (%)	24 (10.0)	33 (13.8)	0.204	13 (9.0)	21 (14.5)	0.144	24 (8.4)	42 (14.7)	0.018*
Postoperative LOS, median (IQR)	14 (11-19)	14 (11-18)	0.938	13 (11-18)	14 (11-19)	0.609	13 (11-17)	14 (11-20)	0.06
Readmission, n (%)	11 (4.6)	17 (7.1)	0.243	5 (3.4)	8 (5.5)	0.395	16 (5.6)	18 (6.3)	0.724

GLIM, Global Leadership Initiative on Malnutrition; WL, weight loss; BMI, body mass index; SMI, skeletal muscle index; pTNM, pathologic tumor–node–metastasis; PC, postoperative complication; LOS, length of stay; IQR, interquartile range; SD, standard deviation.

*Statistically significant.

### Short-Term Clinical Outcomes

The incidence of major PC in the entire cohort was 25% (297/1,188), of which 9.7% (115/1,188) had severe PC. Univariate and multivariate logistic regression analyses for the risk factors of major and severe PC were conducted in the entire cohort. The results showed that age, low SMI, albumin level, and Charlson score ≥2 were independent risk factors for major and severe PC (all *P <*0.05, **Table 3**).

**Table 3:** Univariate and multivariate logistic regression analysis for postoperative complications in the entire cohort.

Factors	Major PC Univariate		Major PC Multivariate		Severe PC Univariate		Severe PC Multivariate	
	HR (95% CI)	*P* value	HR (95% CI)	*P* value	HR (95% CI)	*P* value	HR (95% CI)	*P* value
Age, years	1.044 (1.030-1.059)	<0.001*	1.028 (1.013-1.044)	<0.001*	1.042 (1.021-1.063)	<0.001*	1.023 (1.001-1.046)	0.039*
Sex, Male/Female	1.107 (0.756-1.369)	0.910			1.044 (0.674-1.618)	0.846		
GLIM-high WL	1.298 (0.947-1.780)	0.105			1.674 (1.087-2.577)	0.019*		
GLIM-low BMI	1.838 (1.286-2.628)	0.001*			1.880 (1.157-3.054)	0.011*		
GLIM-low SMI	1.855 (1.408-2.444)	<0.001*	1.489 (1.110-1.997)	0.008*	1.937 (1.309-2.866)	0.001*	1.536 (1.015-2.326)	0.042*
Hemoglobin, g/L	0.990 (0.985-0.996)	<0.001*			0.994 (0.986-1.002)	0.141		
Albumin, g/L	0.929 (0.903-0.956)	<0.001*	0.958 (0.929-0.988)	0.007*	0.924 (0.888-0.962)	<0.001*	0.950 (0.909-0.992)	0.020*
Charlson score								
1/0	1.485 (1.083-2.037)	0.014*	1.367 (0.986-1.894)	0.061	1.420 (0.891-2.263)	0.140	1.328 (0.825-2.137)	0.243
≥2/0	2.225 (1.586-3.122)	<0.001*	1.902 (1.339-2.702)	<0.001*	2.126 (1.324-3.414)	0.002*	1.838 (1.130-2.989)	0.014*
Tumor size, cm	1.101 (1.041-1.164)	0.001*			1.099 (1.015-1.189)	0.020*		
pTNM stage								
II/I	1.854 (1.291-2.661)	0.001*			1.814 (1.056-3.113)	0.031*		
III/I	1.577 (1.157-2.149)	0.004*			1.727 (1.083-2.755)	0.022*		

PC, postoperative complication; GLIM, Global Leadership Initiative on Malnutrition; WL, weight loss; BMI, body mass index; SMI, skeletal muscle index; pTNM, pathologic tumor–node–metastasis.

^*^Statistically significant.

After PSM analysis, the postoperative LOS and readmission rates among the groups in the three matched cohorts were not statistically significant (all *P >*0.05, **Table 2**). In addition, the PC rates in the matched low SMI group were significantly higher than those in the normal SMI group (major: 31.2% vs. 23.9%, *P* =0.049; severe: 14.7% vs. 8.4%, *P* =0.018), while there was no statistical difference in the matched WL and BMI cohorts (all *P >*0.05, **Table 2**). However, in terms of specific complications, no statistical difference was observed between the low and normal SMI groups (all *P >*0.05, **Table S1**).

### Long-Term Survival Outcomes

The median follow-up period was 49.2 (range: 0.5–81.5) months. The three-year OS and DFS rates in the entire cohort were 67.5% and 64.2%, respectively. Univariate and multivariate Cox regression analyses for the risk factors of OS and DFS rates were performed in the entire cohort. The analysis results showed that age, high WL, tumor size, and TNM stage were independent risk factors for OS and DFS rates (all *P <*0.05, **Table 4**).

**Table 4:** Univariate and multivariate Cox regression analysis for OS and DFS in the entire cohort.

Factors	OS Univariate		OS Multivariate		DFS Univariate		DFS Multivariate	
	HR (95% CI)	*P* value	HR (95% CI)	*P* value	HR (95% CI)	*P* value	HR (95% CI)	*P* value
Age, years	1.038 (1.028-1.049)	<0.001*	1.035 (1.024-1.045)	<0.001*	1.032 (1.022-1.042)	<0.001*	1.028 (1.018-1.038)	<0.001*
Sex, Male/Female	1.327 (1.046-1.684)	0.020*			1.287 (1.026-1.614)	0.029*		
GLIM-high WL	2.011 (1.619-2.499)	<0.001*	1.327 (1.063-1.656)	0.012*	1.967 (1.595-2.425)	<0.001*	1.283 (1.036-1.589)	0.022*
GLIM-low BMI	1.608 (1.240-2.085)	<0.001*			1.562 (1.214-2.009)	0.001*		
GLIM-low SMI	1.475 (1.201-1.812)	<0.001*			1.402 (1.150-1.710)	0.001*		
Hemoglobin, g/L	0.991 (0.988-0.995)	<0.001*			0.991 (0.987-0.995)	<0.001*		
Albumin, g/L	0.930 (0.912-0.949)	<0.001*			0.934 (0.916-0.952)	<0.001*		
Charlson score								
1/0	1.223 (0.970-1.541)	0.088			1.159 (0.926-1.450)	0.198		
≥2/0	1.218 (0.932-1.592)	0.148			1.213 (0.940-1.566)	0.138		
Tumor size, cm	1.240 (1.195-1.286)	<0.001*	1.106 (1.056-1.158)	<0.001*	1.237 (1.194-1.282)	<0.001*	1.105 (1.057-1.155)	<0.001*
pTNM stage								
II/I	2.536 (1.734-3.708)	<0.001*	1.777 (1.198-2.637)	0.004*	2.431 (1.699-3.477)	<0.001*	1.760 (1.214-2.553)	0.003*
III/I	7.166 (5.245-9.791)	<0.001*	5.050 (3.612-7.062)	<0.001*	6.845 (5.110-9.171)	<0.001*	4.893 (3.569-6.708)	<0.001*

OS, overall survival; DFS, disease−free survival; GLIM, Global Leadership Initiative on Malnutrition; WL, weight loss; BMI, body mass index; SMI, skeletal muscle index; pTNM, pathologic tumor–node–metastasis.

^*^Statistically significant.

**Figure 2:**
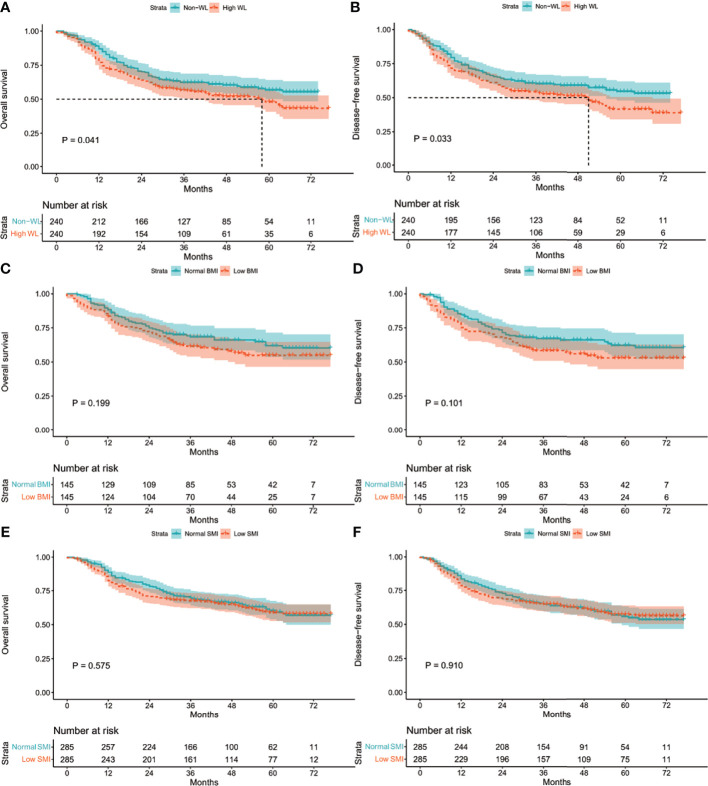
Kaplan-Meier curves: **(A)** in the matched WL cohort for OS rate; **(B)** in the matched WL cohort for DFS rate; **(C)** in the matched BMI cohort for OS rate; **(D)** in the matched BMI cohort for DFS rate; **(E)** in the matched SMI cohort for OS rate; **(F)** in the matched SMI cohort for DFS rate. WL, weight loss; BMI, body mass index; SMI, skeletal muscle index; OS, overall survival; DFS, disease-free survival.

The Kaplan–Meier curves in the entire cohort showed that high WL, low BMI, and low SMI were associated with poor OS and DFS rates (all *P <*0.001, **Figure S1**). After matching, high WL was still associated with poor OS and DFS rates (*P* =0.041; *P* =0.033, **Figures 2A–D**), while low BMI and low SMI were not (*P* =0.575; *P* =0.910, **Figures 2E, F**). In addition, the median follow-up for the WL-matched cohort was 49.0 (range: 0.5–81.5) months. The three-year OS and DFS rates in the WL-matched cohort were 55.0% and 53.0%, respectively.

## Discussion

Malnutrition is a public health problem often associated with a poor prognosis in patients with gastric malignancies. Early recognition of malnutrition and active nutritional support measures can improve the nutritional status and clinical outcomes of patients with GC (6, 23). Malnutrition has recently attracted the attention of clinicians as a risk factor that may be corrected. The GLIM recently published new standard guidelines for diagnosing malnutrition and have encouraged independent verification and research on these standard guidelines in specific populations (8). For the first time, the current study used the PSM analysis method was used to explore the correlation between the three phenotype-based criteria of GLIM and the short- and long-term prognosis of patients with GC.

It is well known that malnourished patients often present with worse nutritional reserve indicators, such as hemoglobin and serum albumin levels (2, 9). In addition, elderly patients are more prone to suffer from malnutrition, sarcopenia, and weakness due to weakened body functions, organ decline, and reduced immunity (24). GC is a highly aggressive form of cancer, especially in the advanced or metastatic stage, and the symptoms of reduced nutritional intake and absorption and gastrointestinal obstruction were more obvious than in other cancer patients (23, 25). Considering that the clinical baselines of malnourished patients were worse than those of well-nourished patients, and the associated adverse outcomes were unsurprising and obvious, consistent with the findings in this study. However, it was hard to implement a randomized controlled trial to explore the correlation between the components of the GLIM criteria and the prognosis of GC patients. Therefore, this study adopted the PSM analysis method to control the bias to the greatest extent possible.

A history of WL is an essential part of nutritional screening and evaluation (4, 6) and has a long history of use in the clinical assessment of malnutrition. It has been reported that high WL in GLIM criteria was significantly related to severe PC and mortality following major abdominal surgery (12). In the matched cohort of this study, high WL was also associated with poor survival prognosis, but not with major and severe PCs. Therefore, we inferred that cut-off value of the high WL in GLIM criteria has a better predictive ability on survival prognosis of GC, and that the poor clinical baselines of patients with high WL may be the reason for the higher rate of complications. In addition, the weight of patients after gastrectomy is typically reduced by 6–17% (26) and is closely related to poor compliance with postoperative adjuvant chemotherapy. Thus, WL is also an important risk factor for quality of life (QOL) and survival (27, 28).

BMI is usually used to assess nutritional status and is currently the most widely studied body composition parameter. However, preoperative low BMI as an indicator of poor prognosis in GC patients is still controversial (29, 30). BMI was not sufficient to identify all patients facing nutritional challenges, especially in those with increased BMI. It was reported that cancer associated malnutrition was still prevalent among overweight and obese people as defined by BMI (31). Loss of muscle mass may be masked by a high fat mass, making it difficult for BMI to accurately assess actual nutritional and functional status (20, 31). The median BMI of the patients in this study was relatively low, which may be more representative of the Asian population. Therefore, we used the recommended BMI values for Asians reported in the GLIM guidelines that define low BMI as <18.5 kg/m^2^ for patients <70 years old or <20 kg/m^2^ in patients ≥70 years old (8). Before the matching analysis, patients with low BMI were closely related to advanced age, high WL, low SMI, anemia, hypoalbuminemia, high Charlson score, and late tumor stage. Therefore, patients in the low BMI group had more frequent complications and worse long-term prognoses. However, the results of the matching analysis showed that low BMI was not associated with the long- and short-term prognoses of patients with GC. Therefore, further prospective studies are needed to analyze the effectiveness of these criteria in predicting the prognoses of patients with GC.

Abdominal CT imaging has the advantages of accuracy, non-invasiveness, and convenience. It is often used in patients with GC to evaluate tumor staging and monitor treatment during follow-up. Importantly, the accuracy of CT scans in quantifying whole-body skeletal muscle mass can reach 99% (32). Decreased skeletal muscle mass is a key clinical manifestation of cancer-related malnutrition that can lead to the development of sarcopenia (33, 34). A recent meta-analysis reported that low SMI in the L3 cross section is closely related to poor prognosis in GC patients (35). However, there is no consensus on the cut-off value of SMI in the L3 cross section; thus, the current study adopted a recommended cut-off value from our institution’s previous research (22). We found that low SMI was an independent risk factor for complications, rather than long-term survival prognosis, which was consistent with the results of the PSM analysis. Lee et al. found that, compared with preoperative low SMI, postoperative low SMI may be a more important risk factor for long-term survival (34). Although sex had no significant effect on complications and survival, female patients in this study seemed to be more likely to experience low SMI. This may be explained by the sexual dimorphism of skeletal muscle mass that is attributed to differences in hormone levels and gene expression between males and females (36, 37) and can also lead to differences in muscle loss associated with aging.

Exercise and nutritional interventions are critical for maintaining muscle mass and preventing involuntary body weight loss (BWL). Resistance exercise contributes to the synthesis of muscle proteins (38), and the anabolic effect of exogenous amino acids on muscle proteins is enhanced by previous exercise (39). A prospective study by Yamamoto et al. found that some GC patients with sarcopenia can reverse their sarcopenic state by participating in preoperative exercise and nutritional interventions that may improve the postoperative clinical outcome (40). A randomized controlled trial conducted in Japan proposed that daily oral nutritional supplements (ONS) >200 mL after gastrectomy can significantly improve BWL after 1 year (41). In addition, Meng et al. reported that, for patients with nutritional risk after gastrectomy, ONS after discharge can improve nutritional outcomes, skeletal muscle maintenance, chemotherapy tolerance, and some QOL variables (42). These results indicate that preoperative and postoperative supportive measures may improve the clinical outcomes of patients with GC.

The current study has some limitations. First, this study was limited by the retrospective single-center design despite its large sample size, and further multi-regional validation studies are necessary. Second, despite the rigorous PSM analysis, our conclusions were limited by other variables that could not be measured. For example, we did not include preoperative and postoperative nutritional support data in the analysis due to incomplete data collection. Third, this study only analyzed the data of GC patients who underwent radical resection, so the conclusions drawn from this study cannot be generalized to GC patients receiving neoadjuvant chemotherapy or metastatic patients.

In conclusion, high WL was associated with poor OS and DFS rates, and low SMI was associated with high PC rates. However, a low BMI may not be associated with poor clinical outcomes. Of the GLIM criteria, high WL and low SMI may be more valuable in predicting prognosis than low BMI in order to optimize clinical intervention.

## Data Availability Statement

The raw data supporting the conclusions of this article will be made available by the authors, without undue reservation.

## Ethics Statement

The studies involving human participants were reviewed and approved by the Ethics Committee of the First Affiliated Hospital of Wenzhou Medical University. Written informed consent for participation was not required for this study in accordance with the national legislation and the institutional requirements. Written informed consent was not obtained from the individual(s) for the publication of any potentially identifiable images or data included in this article.

## Author Contributions

Y-SH and X-DC contributed to conception and design of the study. L-BX, Y-QC, and W-JC acquired the data. L-BX, S-XZ, and LW analyzed and interpreted data and carried out the statistical analysis. L-BX and T-TM wrote the first draft of the manuscript. All authors contributed to manuscript revision, read, and approved the submitted version.

## Conflict of Interest

The authors declare that the research was conducted in the absence of any commercial or financial relationships that could be construed as a potential conflict of interest.

## Publisher’s Note

All claims expressed in this article are solely those of the authors and do not necessarily represent those of their affiliated organizations, or those of the publisher, the editors and the reviewers. Any product that may be evaluated in this article, or claim that may be made by its manufacturer, is not guaranteed or endorsed by the publisher.
