# Supplementary material for: Correlation Between Components of Malnutrition Diagnosed by Global Leadership Initiative on Malnutrition Criteria and the Clinical Outcomes in Gastric Cancer Patients: A Propensity Score Matching Analysis
Source: Front Oncol. 2022 Mar 3;12:851091. doi: 10.3389/fonc.2022.851091 (PMC8927073; doi:10.3389/fonc.2022.851091)
Supplement: Supplementary file 2 [file Table_1.docx]

**Table S1.** Details of postoperative complications in the propensity score-matched cohort by SMI

|  | All (n=570) | Normal SMI (n=285) | Low SMI (n=285) | *P* value |
| --- | --- | --- | --- | --- |
| Major postoperative complication ^a^ | 157 (27.5) | 68 (23.9) | 89 (31.2) | 0.049* |
| Intra-abdominal infection | 40 (7.0) | 19 (6.7) | 21 (7.4) | 0.743 |
| Pneumonia | 38 (6.7) | 16 (5.6) | 22 (7.7) | 0.314 |
| Anastomotic leakage | 36 (3.2) | 18 (3.2) | 18 (3.2) | 1.000 |
| Pleural effusion | 22 (3.3) | 6 (2.1) | 16 (5.6) | 0.102 |
| Hemorrhage | 20 (3.5) | 7 (2.5) | 13 (4.6) | 0.172 |
| Bowel obstruction | 16 (2.8) | 7 (2.5) | 9 (3.2) | 0.612 |
| Venous thrombosis | 16 (2.8) | 9 (3.2) | 7 (2.5) | 0.612 |
| Gastroparesis | 14 (2.5) | 7 (2.5) | 7 (2.5) | 1.000 |
| Heart failure | 10 (1.8) | 4 (1.4) | 6 (2.1) | 0.523 |
| Hepatic insufficiency | 8 (1.4) | 2 (0.7) | 6 (2.1) | 0.286 |
| Wound infection | 8 (1.4) | 6 (2.1) | 2 (0.7) | 0.286 |
| Respiratory failure | 7 (1.2) | 3 (1.1) | 4 (1.4) | 1.000 |
| Intra-abdominal fluid | 6 (1.1) | 2 (0.7) | 4 (1.4) | 0.686 |
| Renal insufficiency | 5 (0.9) | 0 (0.0) | 5 (1.8) | 0.061 |
| Septicemia | 5 (0.9) | 1 (0.4) | 4 (1.4) | 0.373 |
| Delirium | 4 (0.7) | 1 (0.4) | 3 (1.1) | 0.624 |
| Pancreatic fistula | 3 (0.5) | 3 (1.1) | 0 (0.0) | 0.249 |
| Lymphatic fistula | 3 (0.5) | 3 (1.1) | 0 (0.0) | 0.249 |
| Severe postoperative complication ^b^ | 66 (11.6) | 24 (8.4) | 42 (14.7) | 0.018* |
| Hospital mortality ^c^ | 6 (1.1) | 2 (0.7) | 4 (1.4) | 0.686 |

Abbreviations: SMI, skeletal muscle index.

^a^ Clavien–Dindo grade Ⅱ and above.

^b^ Clavien–Dindo grade Ⅲ and above.

^c^ Clavien–Dindo grade Ⅴ.

* Statistically significant.
